# Annual acknowledgement of manuscript reviewers

**DOI:** 10.1186/1471-2288-13-45

**Published:** 2013-04-05

**Authors:** Adrian Aldcroft

**Affiliations:** 1 BioMed Central, Floor 6, 236 Gray's Inn Road, London, WC1X 8HB, UK

## Abstract

**Contributing reviewers:**

The editors of *BMC Medical Research Methodology* would like to thank all our reviewers who have contributed to the journal in Volume 12 (2012).

## 

Kofi Adragni

USA

Ahmed Akl

Egypt

Igor Akushevich

USA

Rahim Alhamzawi

Uzbekistan

Arthur Allignol

Germany

Gareth Ambler

United Kingdom

Rebecca Armstrong

Australia

Mohsen Asadi-Lari

Iran

Felix Assah

Cameroon

Lorna Aucott

United Kingdom

Michela Baccini

Italy

Vinita Bahl

USA

Connie Bales

USA

Karla Ballman

USA

Gordon Bannister

United Kingdom

Elizabeth Barr

Australia

Maite Barrios

Spain

Xavier Basagana

Spain

John Bass

Australia

Per Bech

Denmark

Betsy Jane Becker

USA

Iris Bell

USA

Ralf Bender

Germany

Vance Berger

USA

Ricardo V Bessa-Nogueira

Brazil

Jakob Bjorner

Denmark

Bronagh Blackwood

United Kingdom

Martin Bland

United Kingdom

Stewart Bond

USA

Marco Bonetti

Italy

Jack Bowden

United Kingdom

Hans Johan Breidablik

Norway

Anna Brettschneider

Germany

Matthias Briel

Switzerland

Sinead Brophy

United Kingdom

Michelle Brown

USA

Valerie Brueton

United Kingdom

Maciej Buchowski

USA

Matthew Burnell

United Kingdom

Janie Busby Grant

Australia

John Campbell

New Zealand

Paolo Capodaglio

Italy

Josep Carrasco

Spain

Joao Andre Carrico

Portugal

Bendix Carstensen

Denmark

Santanu Chakraborty

USA

Karim Chamie

USA

Dung-Tsa Chen

USA

Maggie Chen

Canada

Yi Cheng

USA

Andrea Cherrington

United Kingdom

Gordon Cheung

Hong Kong

Wong Chi Sang Martin

Hong Kong

Rosanna Chianese

Italy

Yasutaka Chiba

Japan

Young Cho

USA

Alexander Clark

Canada

David Clark

USA

Melissa Clark

USA

Rachel Colley

Canada

Gary Collins

United Kingdom

Lyn Colvin

Australia

Warren Comulada

USA

Joel Coste

France

Michael Crowther

United Kingdom

Lea Cupul-Uicab

USA

Desmond Curran

Ireland

Douglas Curran-Everett

USA

Kate Curtis

Australia

Getachew Dagne

USA

Anna Dahl

Sweden

Eleonora Dal Grande

Australia

Hercules Dalianis

Sweden

Hugh Dawkins

Australia

Mark De Belder

United Kingdom

Dinanda De Jager

Netherlands

Wilco De Jager

Netherlands

Isabel De Salis

United Kingdom

Fulvio De Santis

Italy

Rob De Vries

Netherlands

Thomas Debray

Netherlands

Mahshid Dehghan

Canada

Kevin Delucchi

USA

Sheena Derry

United Kingdom

Claus Dethlefsen

Denmark

Shayesta Dhalla

Canada

Paul Dickman

Sweden

Conor Dolan

Netherlands

Deborah Donnell

USA

Diana Doumas

USA

Frances Drummond

Ireland

Sascha Dublin

USA

Alison Eastwood

United Kingdom

Steven Edland

USA

Michelle Edwards

United Kingdom

Brian Egleston

USA

Sarah Emerson

USA

Wilco Emons

Netherlands

Bengt I Eriksson

Sweden

Xiang Fang

USA

Xiangming Fang

USA

Nancy Feeley

Canada

Matthew Ferrari

USA

Lorraine Ferris

Canada

Jodene Fine

USA

Livio Finos

Italy

Mariel Mckenzie Finucane

USA

Marta Fiocco

Netherlands

Nancy Fleischer

USA

Ines Florath

Germany

Francisco Florez-Revuelta

United Kingdom

Gerdine Adriana Johanna Fransen

Netherlands

Tim Friede

Germany

Lin Fritschi

Australia

Birgit Fullerton

Germany

Andrea Furlan

Canada

Anna Gagliardi

Canada

Chris Gale

United Kingdom

Jingjing Gao

USA

Christian Geiser

USA

Oke Gerke

Denmark

Ronald Geskus

Netherlands

Joe Gfroerer

USA

Daniel Gillen

USA

Bruno Giraudeau

France

John Glasser

USA

John Goffin

Canada

Michael Goldacre

United Kingdom

David Goldsbury

Australia

Joanna Goldthorpe

United Kingdom

Miguel Goncalves

United Kingdom

Joao Gonçalves-Pereira

Portugal

Jonathan Graffy

United Kingdom

Patrick Grahaam

New Zealand

Maria Grant

United Kingdom

Rolf Groenwold

Netherlands

Ted Haines

Canada

Helinä Hakko

Finland

Andac Hamamci

Turkey

Ghassan Hamra

USA

Andria Hanbury

United Kingdom

Ron Leonardus Hubertus Handels

Netherlands

Peter Hannan

USA

Karin Hannes

Belgium

Tina Hansen

Denmark

Kirstine Moll Harboe

Denmark

Janet Hardy

Australia

Frank Harrell

USA

Mark Hawley

United Kingdom

Michael Hayn

Germany

Ahmad Hazem

USA

Yulei He

USA

Donald Hedeker

USA

Glenn Heller

USA

Marion Henderson

United Kingdom

Kristien Hens

Netherlands

Michael Hermanussen

Germany

Julian Higgins

United Kingdom

Sebastian Hoffmann

Germany

Joeri Hofmans

Belgium

Will Hopkins

New Zealand

Tanya Horsley

Canada

Keith Horvath

USA

Jeremy Horwood

United Kingdom

David Howard

USA

Marvin Hsiao

Canada

Chiu-Hsieh (Paul) Hsu

USA

Edward Ip

USA

Romaina Iqbal

Pakistan

Afisi Ismaila

Canada

Noah Ivers

Canada

Christopher Jackson

United Kingdom

Mona Jeffreys

United Kingdom

José Manuel Jerez-Aragonés

Spain

Shuang Ji

USA

Timothy Johnson

USA

Judith K Jones

USA

Richard N. Jones

USA

Michele Jonsson Funk

USA

Lydia Kapiriri

Canada

Paul Karanicolas

Canada

Monika Kastner

Canada

Jay Kaufman

Canada

Nicola Kayes

New Zealand

Marilyn Kendall

United Kingdom

Peter R Killeen

USA

Seung-Sup Kim

USA

Young-Il Kim

USA

Peter Kimani

United Kingdom

Alison Kitson

United Kingdom

Mieke Koehoorn

Canada

Inke König

Germany

Alex Kral

USA

Andrew Kramer

USA

Richard Kravitz

USA

Doris Tove Kristoffersen

Norway

Ian Kudel

USA

Yogish Kudva

USA

P. Paul F.M. Kuijer

Netherlands

Pim Kuipers

Australia

Malhar Kumar

India

Yong-Fang Kuo

USA

Maarit Laaksonen

Finland

Peter Lachenbruch

USA

Alicia Lacoma

Spain

Fiona Lampe

United Kingdom

T. Dianne Langford

USA

Klaus Langohr

Spain

Michael Larsen

USA

Tricia Leahey

USA

Claire Leathem

United Kingdom

Karin Leder

Australia

Katherine Jane Lee

Australia

Pamela Leece

Canada

Mariska M. G. Leeflang

Netherlands

Russell Lenth

USA

Lucy Lewis

Australia

Shu Qin Li

Australia

Tianjing Li

USA

Megan Lim

United Kingdom

Hester Floor Lingsma

Netherlands

Shiliang Liu

Canada

Craig Lockwood

Australia

Qi Long

USA

Francisco Louzada

Brazil

Guobing Lu

United Kingdom

Patricia Lucas

United Kingdom

Anna Lugnér

Netherlands

Wei-Ming Luh

Taiwan

Sanja Lujic

Australia

Kate Lyden

USA

Xiaoye Ma

USA

Luciana Macedo

Canada

Anne Magnus

Australia

Markku Mahonen

Finland

Donna Mak

Australia

Laxmaiah Manchikanti

USA

Kenneth Mandl

USA

Ana Marusic

Croatia

Edward Mascha

USA

Elaine Maxwell

United Kingdom

Kathleen Mazor

Uruguay

Sati Mazumdar

USA

Johanna Mazur

Germany

Lawrence Mccandless

Canada

Serena Mccluskey

United Kingdom

Elaine Mccoll

United Kingdom

Marian Mcdonagh

USA

Dean Mckenzie

Australia

Mounir Mesbah

France

Barbara Meyer

Australia

William Mietlowski

USA

Anthony Miller

Canada

Edward Mills

Canada

Niels Christian Moeller

Denmark

Sigrun Mogedal

Norway

Rahim Moineddin

Canada

Karel Moons

Netherlands

Charity Moore

USA

Anne Moseley

Australia

Brendan Mulhern

United Kingdom

Clemma Muller

USA

Keith Muller

USA

Jessica Myers

USA

Florian D. Naal

Switzerland

Dave Nelson

USA

Markus Neuhäuser

Germany

Denise Nishita

USA

Carlos Nordt

Switzerland

Abigail Norris Turner

USA

Peter Nowak

Austria

Maria Nyholm

Sweden

Eiji Oda

Japan

Donna Helene Odierna

USA

Elizabeth Ogburn

USA

Jude Uzoma Ohaeri

Kuwait

Andrew Olshan

USA

Maria Ospina

Canada

Yi Pan

USA

Orestis Panagiotou

Greece

Nikolaos Pandis

Greece

Xavier Paoletti

France

Aliya Pardhan-Ali

Canada

Sang-Won Park

Korea South

Maxine Patel

United Kingdom

Yudi Pawitan

Sweden

Linda Peelen

Netherlands

Isabel Peña-Rey

Spain

Laure Perrier

Canada

Paul Peters

Canada

Morten Aa. Petersen

Denmark

Laurent Pezard

France

Ruth Pfeiffer

USA

Elizabeth Pienaar

South Africa

Hans-Peter Piepho

Germany

Carol Pierannunzi

USA

Rogerio Pinto

USA

Dartagnan Pinto Guedes

Brazil

Wim Pinxten

Belgium

Yannis Pitsiladis

United Kingdom

Robert Platt

Canada

Pierre Pluye

Canada

Maja Pohar Perme

Slovenia

John Powers

USA

Siddharth Pratap

USA

Gabriela Prutsky

USA

Eleanor Pullenayegum

Canada

Yongsong Qin

China

Gilbert Ramirez

United Kingdom

Elisabete Ramos

Portugal

Batool Rashidi

Iran

Elizabeth Reddy

Tanzania

Michael Reichenheim

Brazil

David Rempel

USA

Shiquan Ren

Australia

Benjamin Riche

France

Nicola Ring

United Kingdom

James Robins

United Kingdom

Nikki Rogers

USA

Cecilia Rogmark

Sweden

Angela Rose

Barbados

Carla Rossi

Italy

Jason Roy

USA

Patrick Royston

United Kingdom

Gerta Rücker

Germany

Jorun Rugkasa

United Kingdom

Mark Rutherford

United Kingdom

Emilio Sacchetti

Italy

Ritu Sadana

Switzerland

Mohsen Sadatsafavi

Canada

Georgia Salanti

United Kingdom

Valeria Sambucini

Italy

Cristina Santos

Portugal

Charles Scales

USA

Tom Scheike

Denmark

Roberta Scherer

USA

Heinz Schmidli

Switzerland

Claudia Schueller-Weidekamm

Austria

Kevin Schulman

USA

Stephen Senn

Luxembourg

Larissa Shamseer

Canada

Han Lin Shang

Australia

Shulian Shang

USA

Donald Sharpe

Canada

Lianne Sheppard

USA

Ilya Shpitser

USA

Ian Shrier

Canada

Ghazi Shukur

Sweden

Volkert Siersma

Denmark

Richard Silverwood

United Kingdom

Julius Sim

United Kingdom

David Simel

USA

Iveta Simera

United Kingdom

David Simmons

United Kingdom

Freddy Sitas

Australia

Becky Skidmore

Canada

Merran Smith

Australia

Valerie Smith

Ireland

Niels Smits

Netherlands

Derek Smolenski

USA

Janet Smylie

Canada

Fujian Song

United Kingdom

Helle Sorensen

Denmark

Marc Souris

France

Rodney Sparapani

USA

Donna Spiegelman

USA

Claudia Spies

Germany

Mark Spigt

Netherlands

Renske Spijkerman

Netherlands

Jacqueline Starr

USA

Jay Steingrub

USA

Martijn Steultjens

United Kingdom

Christopher Stevenson

Australia

Ewout Steyerberg

Netherlands

David Stoffer

USA

Elisabeth Strandhagen

Sweden

Kathrin Stucke

Germany

Sibylle Sturtz

Germany

S V Subramanian

USA

Richard Suminski

USA

Sangwoo Tak

USA

Wan Tang

USA

Anne Taylor

Australia

Jean Tchuenche

USA

Michel Tenenhaus

France

Gerben Ter Riet

Netherlands

Marroon Thabane

Canada

Evan Thacker

USA

Carel Thijs

Netherlands

Kristian Thorlund

Canada

Lili Tian

USA

Jennifer Tieman

Australia

Kanae Togo

Japan

Zelda Tomlin

United Kingdom

George Tomlinson

Canada

Allison Tong

Australia

Sven Trelle

Switzerland

Tom Trikalinos

Greece

Chi-Hong Tseng

USA

Alex Tsodikov

USA

Charles Turner

USA

Jb Unger

USA

Werner Vach

Germany

Gerard Van Breukelen

Netherlands

Ann Van Den Bruel

United Kingdom

Tyler Vanderweele

USA

Jonathan Vangeest

USA

Gabriela Vazquez Benitez

USA

Marit B. Veierød

Norway

Akke Vellinga

Ireland

Sumithra Velupillai

Sweden

Yvonne Vergouwe

Netherlands

Albert Vette

Canada

Corrine Voils

USA

Antonio Volpi

Italy

Ljiljana Vuckovic-Dekic

Serbia

Willem Waegeman

Belgium

Elizabeth Wager

United Kingdom

Harald Walach

United Kingdom

Jichuan Wang

USA

Robert Ware

Australia

Eric Weinhandl

USA

Greg Welk

USA

Nicky Welton

United Kingdom

Robert West

United Kingdom

Suzanne West

USA

Cathy Widom

USA

Duminda Wijeysundera

Canada

Kirk Wilhelmus

USA

Andrew Willan

Canada

Brian H Willis

United Kingdom

Emiel Frans Maria Wouters

Netherlands

Hong-Dar Wu

Taiwan

Pei-Chen Wu

Taiwan

Wei Wu

USA

Yukun Wu

USA

Sophie Wuerger

United Kingdom

David Wyllie

United Kingdom

Zhenzhen Xu

USA

Chenglin Ye

Canada

Binbing Yu

USA

Antonia Zapf

Germany

Liz Zelinski

USA

Nanhua Zhang

USA

Song Zhang

USA

Xiaofeng Zhou

USA

Donald Zimmerman

Canada

## Pre-publication history

The pre-publication history for this paper can be accessed here:

http://www.biomedcentral.com/1471-2288/13/45/prepub

